# The predictive value of serum OX-LDL and 25-(OH)-D levels for cerebral edema in patients with hypertensive cerebral haemorrhage

**DOI:** 10.5937/jomb0-62057

**Published:** 2026-05-22

**Authors:** Chao Xu, Bo Wang, Niu Liu, Lei Bao

**Affiliations:** 1 Nanjing Medical University, Nanjing First Hospital, Department of Emergency, Nanjing, China; 2 Chinese Academy of Medical Sciences, Cancer Hospital, Department of Neurosurgery, Beijing, China

**Keywords:** hypertensive cerebral haemorrhage, minimally invasive hematoma evacuation, cerebral oedema, oxidised modified low-density lipoprotein, 25-hydroxyvitamin D, hipertenzivno intracerebralno krvarenje, minimalno invazivna evakuacija hematoma, cerebralni edem, oksidovani lipoprotein niske gustine, 25-hidroksivitamin D

## Abstract

**Background:**

To analyse the predictive value of serum oxidation-modified low-density lipoprotein (ox-LDL) and 25-hydroxyvitamin D [25-(OH)-D] levels before minimally invasive hematoma evacuation in patients with hypertensive intracerebral haemorrhage (H ICH) for cerebral oedema.

**Methods:**

300 patients with HICH in our hospital from August 2022 to August 2024 were selected as research subjects. Patients were divided into a high-ox-LDL group (ox-LDL 50 mg/dL) and a low-ox-LDL group (ox-LDL&lt;50 mg/dL) according to their preoperative ox-LDL levels. The patients were separated into two groups based on the amount of serum 25-(OH)-D: high-level 25-(OH)-D (25-(OH)-D 30 ng/mL) and low-level 25-(OH)-D (25-(OH)-D&lt;30 ng/mL). The incidence of cerebral oedema within 48 hours after surgery was compared among the groups. Following minimally invasive hematoma evacuation in patients with HICH, the predictive value of preoperative serum ox-LDL and 25-(OH)-D levels for cerebral oedema was evaluated using receiver operating characteristic (ROC) curves.

**Results:**

The hematoma volume, fibrinogen level, and incidence of cerebral oedema varied significantly among patients with different serum ox-LDL and 25-(OH)-D levels (P&lt; 0.05). The results of point-biserial correlation analysis revealed that the occurrence of cerebral oedema in HICH patients was positively correlated with the serum ox-LDL level (r= 0.455, P&lt; 0.05) and negatively correlated with the serum 25-(OH)-D level (r= -0.534, P&lt; 0.05). Pearson correlation analysis revealed a negative correlation between serum ox-LDL and 25-(OH)-D (r= -0.444, P&lt; 0.05). The areas under the curve (AUCs) for preoperative serum ox-LDL, 25-(OH)-D alone, and combined detection in predicting cerebral oedema following minimally invasive hematoma evacuation in patients with HICH were 0.777, 0.768, and 0.839, respectively, according to ROC analysis. The AUC of combined detection was the largest.

**Conclusions:**

Before minimally invasive hematoma evacuation in patients with HICH, the serum ox-LDL level was high, and the 25-(OH)-D level was low. Preoperative serum ox-LDL and 25-(OH)-D levels have high predictive value for cerebral oedema after minimally invasive hematoma evacuation in patients with HICH.

## Abstract (sr)

**Uvod:**

Cilj se analiziraju prediktivne vrednosti nivoa serumskog oksidacijom modifikovanog lipoproteina niske gustine (ox-LDL) i 25-hidroksivitamina D [25-(OH)-D] pre minimalno invazivne evakuacije hematoma kod pacijenata sa hipertenzivnim intracerebralnim krvarenjem (HICH) kod cerebralnog edema.

**Metode:**

U istraživanje je uključeno 300 pacijenata sa HICH hospitalizovanih u našoj ustanovi u periodu od avgusta 2022. do avgusta 2024. godine. Pacijenti su podeljeni u grupu sa visokim nivoom ox-LDL-a (ox-LDL 50 mg/dL) i grupu sa niskim nivoom ox-LDL-a (ox-LDL&lt;50 mg/dL), prema preoperativnom nivou serumskog ox-LDL-a. Takođe su podeljeni u grupu sa visokim nivoom 25-(OH)-D (30 ng/mL) i grupu sa niskim nivoom 25-(OH)-D (&lt;30 ng/mL). Upoređivana je incidencija cerebralnog edema u roku od 48 sati nakon operacije između grupa. Prediktivna vrednost preoperativnih nivoa serumskog ox-LDL-a i 25-(OH)-D za pojavu cerebralnog edema procenjena je pomoću ROC krive.

**Rezultati:**

Zapremina hematoma, nivo fibrinogena i učestalost cerebralnog edema značajno su se razlikovali među pacijentima sa različitim nivoima serumskog ox-LDL-a i 25-(OH)-D (P&lt; 0,05). Analiza "point-biserial" korelacije pokazala je da je pojava cerebralnog edema kod pacijenata sa HICH pozitivno korelisana sa nivoom serumskog ox-LDL-a (r= 0,455, P&lt; 0,05), a negativno korelisana sa nivoom serumskog 25-(OH)-D (r= -0,534, P&lt; 0,05). Pirsonova korelaciona analiza pokazala negativnu korelaciju između nivoa ox-LDL-a i 25-(OH)-D (r=-0,444, P&lt; 0,05). Površina ispod krive (AUC) za predikciju cerebralnog edema nakon minimalno invazivne evakuacije hematoma pomoću preoperativnih nivoa ox-LDL-a, 25-(OH)-D pojedinačno i njihove kombinovane analize je iznosila 0,777, 0,768 i 0,839, redom. Najveću AUC vrednost imala je kombinovana analiza.

**Zaključak:**

Kod pacijenata sa HICH, pre minimalno invazivne evakuacije hematoma, nivo serumskog ox-LDL-a je povišen, dok je nivo 25-(OH)-D snižen. Preoperativni nivoi serumskog ox-LDL-a i 25-(OH)-D imaju visoku prediktivnu vrednost za pojavu cerebralnog edema nakon minimalno invazivne evakuacije hematoma kod ovih pacijenata.

## Introduction

At present, minimally invasive hematoma evacuation is widely used in clinical practice to treat hypertensive intracerebral haemorrhage (HICH), as it can promptly remove the hematoma and reduce the damage it causes to surrounding brain tissue [1]. A typical side effect following minimally invasive hematoma evacuation surgery is cerebral oedema; the prognosis and postoperative disease progression of individuals with HICH are significantly impacted [2]. In severe cases, it can induce intracranial hypertension and aggravate the degree of brain tissue damage, resulting in a poor prognosis for patients. Low-density lipoprotein is a common type of lipid substance that contains a large amount of unsaturated fatty acids [3][4][5]. It is prone to self-oxidation and the formation of oxidised, modified low-density lipoprotein (ox-LDL), which can induce various pathological and physiological reactions, such as oxidative stress and inflammation. However, at present, there is still insufficient evidence on whether serum ox-LDL and 25-(OH)-D can predict cerebral oedema after minimally invasive hematoma removal in patients with HICH [6]. Hypertensive cerebral haemorrhage (HICH) is a serious cerebrovascular event that leads to high fatality and disability rates. Its poor prognosis is related mainly to secondary cerebral oedema. At present, in clinical practice, effective early prediction methods for the risk of cerebral oedema remain insufficient and rely primarily on imaging examinations, which are often delayed. The need to explore more sensitive and convenient biological markers to guide early intervention is urgent [7]. Oxidative stress plays a core role in secondary injuries after cerebral haemorrhage. A significant byproduct of oxidative stress, elevated levels of oxidised low-density lipoprotein (ox-LDL) are linked to vascular endothelial damage, disruption of the blood-brain barrier, and heightened neuroinflammatory responses, all of which can worsen cerebral oedema [8]. Moreover, as a hormone with neuroprotective potential, low levels of its active form [9]. Vitamin D may mitigate oxidative stress and inflammation, thereby preserving the blood-brain barrier. Still, its specific predictive value in the development of brain oedema after HICH has not yet been clarified [10][11][12].

To explore whether the levels of serum ox-LDL and 25-(OH)D can serve as reliable biological indicators for predicting the risk of brain oedema in patients with HICH. Evaluating the predictive value of combining these two indicators has significant clinical implications. It is expected to provide a new theoretical basis for early identification of high-risk patients, the formulation of precise intervention strategies, and improvements in patient prognosis.

## Materials and methods

### General information

In this prospective cohort study, 300 HICH patients treated at our hospital between August 2022 and August 2024 were selected to participate.

Inclusion criteria: (1) met the diagnostic criteria for HICH in the International Journal of Neurology (9th Edition); (2) aged 18-62 years; and (3) admitted to the hospital 6 hours after the onset of the disease and treated with minimally invasive hematoma evacuation.

Exclusion criteria: (1) cerebral haemorrhage caused by ruptured intracranial aneurysms, venous malformations, etc.; (2) comorbid malignant diseases of the haematological system; (3) complicated with infectious diseases such as hepatitis and AIDS; (4) combined liver and kidney function impairment; and (5) long-term use of anticoagulant drugs. Patients were divided into a high-ox-LDL group (ox-LDL 50 μg/dL) and a low-ox-LDL group (ox-LDL<50 μg/dL) according to their preoperative ox-LDL levels. Additionally, according to the serum 25(OH) D levels, patients were divided into high-level 25(OH) D groups (25(OH) D acuity of 30 ng/mL) and low-level 25(OH) D groups [25(OH)-D<30 ng/mL].

### Diagnostic methods for cerebral oedema

The diagnosis of cerebral oedema was made in accordance with the »International Surgery (9th Edition)«. A magnetic resonance imaging (MRI) test was performed on each patient 48 hours following the procedure. If the MRI shows a high signal on T2WI and a low signal on T1WI, or if cerebral haemorrhage and mixed signals are present, cerebral oedema can be diagnosed.

### Laboratory index detection

Each patient had 10 mL of peripheral venous blood from the elbow drawn before the procedure, which was then split between two test tubes. One of the samples was placed in a centrifuge for centrifugation (centrifugation speed, 2,500 r/min; centrifugation radius, 15 cm). After centrifugation, the serum was collected, and the levels of ox-LDL and 25-(OH)-D were measured by chemiluminescence and an enzyme-linked immunosorbent assay, respectively (all kits purchased from Beijing Zhengdan International Technology Co., Ltd.). The amounts of aspartate aminotransferase, alanine aminotransferase, and total bilirubin, uric acid and cystatin C in the serum were detected via an automatic biochemical analyser (Henan Xianbang Technology Co., Ltd., model SHIB BH20). Neutrophil count and haemoglobin level via a fully automatic blood cell analyser (Qingdao Hantang Biotechnology Co., Ltd., model: HT-5000). The prothrombin time (PT), activated partial thromboplastin time (APTT), thrombin time (TT), and fibrinogen were detected by a coagulation analyser (Guangxi Biaojia Biotechnology Co., Ltd., model: S2000). Random blood glucose levels were measured with a blood glucose meter (Suzhou Erda Medical Equipment Co., Ltd., model HGM-111). In addition, random urine samples from the patients were collected and analysed using an automatic urine analyser (Guilin URIT Medical Electronics Co., Ltd., model URIT-1550).

### Statistical analysis methods

SPSS 25.0 statistical software was used for data processing and statistical analysis. The independent-samples t-test was used for group comparisons, and x̄±s represent normally distributed data. The χ^2^ test was used to compare groups, and count data are presented as percentages or counts. Point-biserial correlation analysis was used to analyse the relationships among serum ox-LDL levels, 25-(OH)-D levels and cerebral oedema after minimally invasive hematoma evacuation in patients with HICH. An investigation of the relationship between serum ox-LDL and 25-(OH)-D levels was conducted using Pearson correlation analysis. Following minimally invasive hematoma evacuation in patients with HICH, the predictive value of serum ox-LDL and 25-(OH)-D levels for cerebral oedema was evaluated using a receiver operating characteristic (ROC) curve. An area under the curve (AUC)>0.90 indicated a high predictive value, and 0.71-0.90 indicated a moderate predictive value. A predictive value of 0.50-<0.71 indicates poor predictive ability.

## Results

### Grouping of HICH patients

Three hundred patients with HICH were divided into a high-ox-LDL group (132 patients) and a low-ox-LDL group (168 patients) based on their serum ox-LDL levels. The ox-LDL levels in the two groups were 55.63±5.60 and 47.12±3.86 μg/dL, respectively. A high-level 25-(OH)-D group of 180 patients and a low-level 25-(OH)-D group of 120 patients were created based on the patients' serum 25-(OH)-D levels. The two groups' 25-(OH)-D levels were 34.02±3.72 and 26.42±3.05 ng/mL, respectively. The patients were split into two groups: those with high levels of oxidised low-density lipoprotein (ox-LDL) (50 μg/dL) and those with low levels (<50 μg/dL), with the cutoff being set at 50 mg/dL. Using 120 ng/mL as the limit of serum 25-hydroxyvitamin D [25-(OH)-D], patients were divided into high-25-(OH)-D groups (30 ng/mL) and low-25-(OH)-D groups (<30 ng/mL). The analysis revealed that among groups with different levels of serum ox-LDL and 25-(OH)-D, significant differences were observed in hematoma volume, fibrinogen levels, and the incidence of cerebral oedema 48 hours after surgery (P<0.05).

### Comparison of the baseline data and laboratory indicators of patients with different serum ox-LDL and 25-(OH)-D levels

There was a statistically significant difference in hematoma volume and fibrinogen level among patients with different serum ox-LDL and 25-(OH)-D levels (P<0.05). Other baseline statistics and laboratory markers did not differ statistically significantly across patients with varying blood ox-LDL and 25-(OH)-D levels (P>0.05), see Table 1 and Table 2.

**Table 1:** Comparison of baseline data and laboratory indicators of patients with different serum ox LDL levels.

Group	n	Gender		Age<br>(years)	Bleeding site				Body mass<br>number<br>(kg/m^2^)	Hematoma<br>volume<br>(mL)
		Male	Female		Basal<br>ganglia	Thalamus	Brain<br>stem	Other		
High-level<br>ox LDL<br>group	132	68	64	58.42±4.66	60	32	24	16	25.08±2.34	16.52±5.46
Low-level<br>ox LDL<br>group	168	96	72	57.83±4.70	80	44	32	12	25.22±2.30	12.03±5.15
x^2^/t		0.236		0.543	0.656				-0.26	3.658
P		0.627		0.589	0.941				0.796	<0.001
Group	n	History of<br>smoking and<br>drinking	Comorbidity			Total<br>bilirubin<br>(μ mol/L)	Alanine<br>aminotransferase<br>(U/L)		Aspartate amino acid<br>Transferase enzyme<br>(U/L)	
			Diabetes	Hyperlipidemia	Obstructive<br>sleep apnea					
High-level<br>ox LDL<br>group	132	52	24	20	12	10.28±3.42	31.42±4.24		29.65±4.18	
Low-level<br>ox LDL<br>group	168	60	40	32	16	10.05±3.38	30.47±4.30		30.15±4.23	
x^2^/t		0.107	0.349	0.196	0.113	0.291	0.955		-0.501	
P		0.744	0.555	0.658	0.737	0.772	0.342		0.618	
Group	n	Uric acid<br>(μmol/L)	Cystatin C<br>(mg/L)	Blood<br>creatinine<br>(μmol/L)	Urea<br>nitrogen<br>(mmol/L)	Red blood<br>cell count<br>(x10^12^/L)	White blood<br>cell count<br>(x10^9^/L)		Platelet count<br>(x10^9^/L)	
High-level<br>ox LDL<br>group	132	254.75±20.38	1.22±0.17	78.85±5.02	5.62±1.06	6.02±1.52	10.24±2.33		165.67±18.65	
Low-level<br>ox LDL<br>group	168	251.85±20.30	1.18±0.20	76.75±4.83	5.66±1.01	5.96±1.47	10.50±2.37		162.67±19.34	
x^2^/t		0.613	0.709	1.845	-0.207	0.201	-0.456		0.677	
P		0.542	0.481	0.069	0.837	0.841	0.650		0.500	
Group	n	Neutrophil count<br>(x10^9^/L)	Haemoglobin<br>(g/L)	PT(s)	APTT(s)	TT(s)	Fibrinogen<br>(ng/L)		Random blood<br>glucose (mmol/L)	
High-level<br>ox LDL<br>group	132	4.38±1.20	132.52±23.56	12.46±0.82	32.85±4.52	17.66±0.62	3.76±0.52		8.24±2.02	
Low-level<br>ox LDL<br>group	168	4.30±1.25	130.88±22.89	12.50±0.84	33.10±4.56	17.62±0.58	3.02±0.60		8.28±1.96	
x^2^/t		0.314	0.304	-0.178	-0.246	0.289	5.608		-0.086	
P		0.754	0.762	0.859	0.806	0.773	<0.001		0.932	

**Table 2:** Comparison of baseline data and laboratory indicators of patients with different serum 25-(OH)-D levels.

Group	n	Gender		Age<br>(years)	Bleeding site				Body mass<br>number<br>(kg/m^2^)	Hematoma<br>volume<br>(mL)
		Male	Female		Basal ganglia	Thalamus	Brain stem	Other		
Low-level<br>25- (OH)<br>- D group	120	64	56	58.90±4.70	64	28	20	8	25.23±2.28	16.30±5.28
High-level<br>25- (OH)<br>- D group	180	100	80	57.24±4.75	92	44	24	20	25.76±2.32	13.53±5.24
x^2^/t		0.036		1.485	0.601				-0.978	2.226
P		0.85		0.142	0.949				0.331	0.029
Group	n	History of<br>smoking and<br>drinking	Comorbidity			Total<br>bilirubin	Alanine<br>aminotransferase<br>(U/L)		Aspartate amino acid<br>Transferase enzyme<br>(U/L)	
			Diabetes	Hyperlipidemia	Obstructive<br>sleep apnea					
Low-level<br>25- (OH)<br>- D group	120	48	20	16	12	9.88±3.40	30.66±4.32		30.22±4.25	
High-level<br>25- (OH)<br>- D group	180	64	36	24	20	10.13±3.38	29.84±4.35		29.75±4.22	
x^2^/t		0.152	0.132	0.12	0.053	-0.313	0.803		0.462	
P		0.697	0.717	0.729	0.819	0.755	0.425		0.646	
Group	n	Uric acid<br>(μmol/L)	Cystatin C<br>(mg/L)	Blood<br>creatinine<br>(μmol/L)	Urea<br>nitrogen<br>(mmol/L)	Red blood<br>cell count<br>(x10^12^/L)	White blood cell<br>count<br>(x10^9^/L)		Platelet count<br>(x10^9^/L)	
Low-level<br>25- (OH)<br>- D group	120	252.68±20.40	1.20±0.19	77.48±4.93	5.70±1.03	8.87±1.50	10.52±2.40		163.89±19.02	
High-level<br>25- (OH)<br>- D group	180	252.08±20.32	1.23±0.22	76.52±4.86	5.73±1.00	5.64±1.55	10.32±2.42		164.02±18.87	
x^2^/t		0.127	-0.793	0.825	-0.127	0.636	0.351		-0.031	
P		0.899	0.430	0.412	0.900	0.527	0.727		0.975	
Group	n	Neutrophil count<br>(x10^9^/L)	Haemoglobin<br>(g/L)	PT(s)	APTT(s)	TT(s)	Fibrinogen<br>(ng/L)		Random blood glucose<br>(mmol/L)	
Low-level<br>25- (OH)<br>- D group	120	4.33±1.22	131.60±23.06	12.42±0.88	32.48±4.53	17.46±0.58	3.80±0.56		8.30±2.12	
High-level<br>25- (OH)<br>- D group	180	4.36±1.26	130.48±22.49	12.53±0.82	33.20±4.57	17.70±0.55	2.98±0.52		8.22±1.98	
x^2^/t		-0.102	0.207	-0.539	0.663	-0.850	6.506		0.17	
P		0.919	0.836	0.591	0.510	0.068	<0.001		0.866	

The baseline data and laboratory indicators of patients with hypertensive intracerebral haemorrhage (HICH) with different preoperative serum levels of oxidised low-density lipoprotein (ox-LDL) and 25-hydroxyvitamin D [25-(OH)-D] were compared and analysed. The results revealed significant differences in key indicators among patients with different serum ox-LDL levels (high/low-level group) and different 25-(OH)-D levels (high/low-level group) (P<0.05). The patient groups with different levels of serum ox-LDL and 25-(OH)-D showed significant intergroup differences in hematoma volume, fibrinogen levels, and the incidence of cerebral oedema within 48 hours after surgery.

### Incidence of cerebral oedema in patients with different levels of ox-LDL and 25 (OH)-D

The incidence of cerebral oedema in the high-level ox-LDL group was 51.52% (68/132), which was greater than the 11.90% (20/168) reported in the low-level ox-LDL group, and the difference was statistically significant (x^2^ = 13.988, P<0.05). In Group D, with low levels of 25(OH), the incidence of cerebral oedema was 53.33% (64/120), which was higher than that in Group D (24/180), and 13.33% of the differences were statistically significant (chi-square=13.894, P<0.05).

Patients with varying preoperative serum ox-LDL levels (high/low level group) and 25-(OH)-D levels (high/low level group) had a statistically significant difference in the incidence of postoperative cerebral oedema (P<0.05). While the incidence of cerebral oedema was significantly lower in the group with high serum 25-(OH)-D levels than in the group with low levels, it was considerably higher in the group with high serum ox-LDL levels than in the group with low levels. Correlation analysis further confirmed that postoperative cerebral oedema was significantly positively correlated with the serum ox-LDL level (r=0.455, P<0.05), and the serum 25-(OH)-D level was substantially inversely correlated with it (r=-0.534, P<0.05).

### Relationships between serum ox-LDL and 25-(OH)-D levels and cerebral oedema

According to the findings of the point-biserial correlation analysis, cerebral oedema in HICH patients was associated with negative (r = -0.534, P < 0.05) and positive (r = 0.455, P < 0.05) correlations with the serum 25-(OH)-D level. According to the Pearson correlation analysis, there was a negative association between serum ox-LDL and 25-(OH)-D levels (r =-0.444, P<0.05).

### Predictive value of serum ox-LDL and 25-(OH)-D levels for cerebral oedema

**Figure 1:**
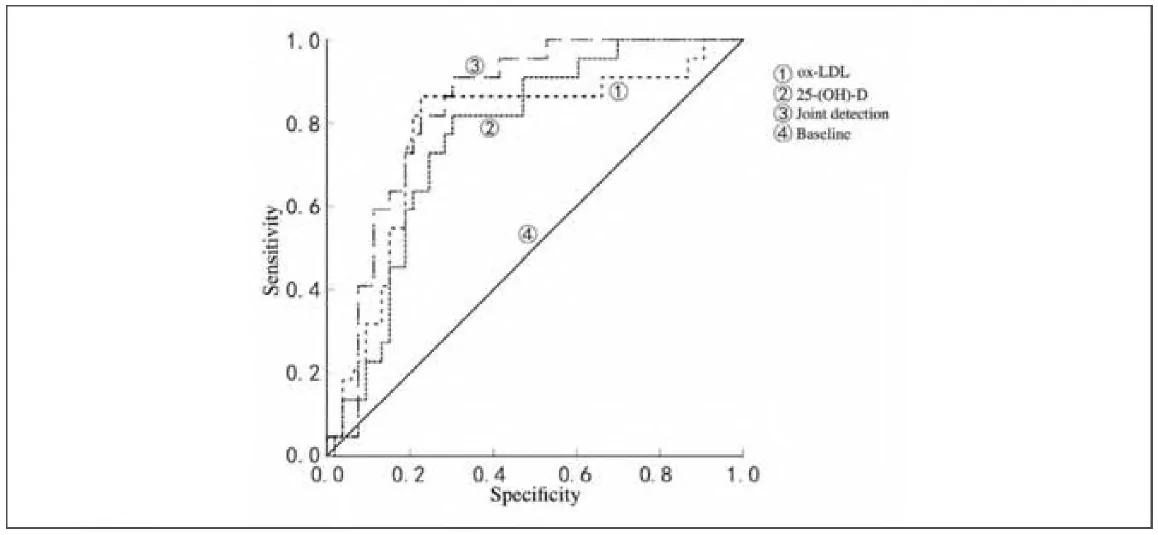
ROC curves of serum ox-LDL and 25-(OH)-D levels for predicting brain oedema.

The areas under the curve (AUCs) for blood ox-LDL and 25-(OH)-D alone and in combination for predicting cerebral oedema in patients with HICH were 0.777, 0.768, and 0.839, respectively, according to ROC analysis. The AUC of combined detection was the highest, see Table 3 and Figure 1.

**Table 3:** The predictive value of serum ox LDL and 25- (OH) - D levels for cerebral oedema.

Item	AUC	AUC 95%CI	P	Cutoff value	Specificity	Sensitivity	Youden index
Serum ox LDL	0.777	0.653~0.901	<0.001	52.860 μg/dL	0.792	0.773	0.565
Serum 25- (OH) - D	0.768	0.659~0.877	<0.001	28.350 ng/L	0.755	0.682	0.437
Joint tests	0.839	0.749~0.929	<0.001	-	0.774	0.818	0.592

Preoperative serum levels of oxidised low-density lipoprotein (ox-LDL) and 25-hydroxyvitamin D [25-(OH)-D] were evaluated for their predictive ability for postoperative cerebral oedema in patients with hypertensive intracerebral haemorrhage (HICH) using ROC curve analysis. The preoperative serum ox-LDL level alone had an area under the curve (AUC) of 0.777 for predicting brain oedema, whereas the AUC of the serum 25-(OH)-D level alone was 0.768, indicating that both had moderate to high predictive values. Notably, when ox-LDL is combined with 25-(OH)-D, its predictive efficacy is significantly improved, with an AUC of 0.839. The occurrence of cerebral oedema was significantly positively correlated with the serum ox-LDL level (r=0.455, P<0.05) and significantly negatively correlated with the 25-(OH)-D level (r=-0.534, P<0.05).

## Discussion

Cerebral oedema usually occurs within 1 to 2 hours after cerebral haemorrhage, reaches its peak at approximately 48 hours, and gradually subsides after 3 to 5 days [13]. The occurrence of cerebral oedema can further aggravate the degree of neurological function impairment in patients with HICH. If not treated in time, it can induce complications such as consciousness disorders and brain herniation, which can have an adverse impact on the prognosis of patients [14][15][16]. At present, CT and MRI remain the main methods for diagnosing cerebral oedema after HICH surgery. Clinical data show that the condition of HICH patients is prone to change during the acute phase, whereas imaging examinations can reflect only the progression of the disease within a certain period and cannot monitor the patient's condition in real time [17]. Serum cytokine detection has many advantages, including rapidity, ease of operation, and high repeatability. Elevated serum ox-LDL levels can mediate vascular endothelial injury and induce cerebral vasospasm, thereby increasing the risk of cerebrovascular disease. The active form of vitamin D, serum 25-(OH)-D, can promote monocyte differentiation into macrophages and inhibit the release of inflammatory cells [18][19][20]. At present, clinical studies on serum ox-LDL and 25-(OH)-D have focused mostly on atherosclerosis, while few reports have examined the relationships between serum ox-LDL and 25-(OH)-D and cerebral oedema after minimally invasive hematoma evacuation in patients with HICH [21].

Cerebral oedema was substantially more common in the high-level ox-LDL and low-level 25-(OH)-D groups than in the low-level ox-LDL and high-level 25-(OH)-D groups (P<0.05). Further analysis revealed that serum ox-LDL and 25-(OH)-D were closely associated with the development of cerebral oedema in patients with HICH [22]. The possible mechanisms by which serum ox-LDL and 25-(OH)-D are related to the occurrence of cerebral oedema are as follows: (1) ox-LDL can bind to its receptor, increase the number of intracellular reactive oxygen species, and reduce the utilisation rate of nitric oxide, thereby inhibiting vasodilation and inducing vascular dysfunction, aggravating the degree of cerebral vasospasm, and increasing the incidence of cerebral oedema [23][24][25]. Moreover, ox-LDL is phagocytosed by macrophages and smooth muscle cells, which can form foam cells, triggering a series of inflammatory responses and disrupting the integrity of microvessels in the tissues surrounding the hematoma, thereby causing cerebral oedema. (2) During the course of disease progression, patients with HICH may experience varying degrees of oxidative stress and inflammatory responses, which can aggravate the degree of brain injury and promote the occurrence and development of brain oedema. By acting on the vitamin D receptor in immune cells, 25-(OH)-D can increase IL-10 activity and decrease the release of inflammatory mediators such as IL-6 and tumour necrosis factor-α, thereby protecting brain tissue and exerting antiinfection effects. Moreover, 25-(OH)-D can also increase-glutamyl transferase levels in brain astrocytes, thereby preventing cerebral oedema by boosting the activities of catalase and superoxide dismutase and preventing oxygen overload [26]. This study suggests that 25-(OH)-D is closely associated with brain oedema in patients with HICH. ROC curve analysis revealed that both individual and combined detection of serum ox-LDL and 25-(OH)-D had predictive value for brain oedema in patients with HICH. Moreover, the best predictive value was obtained when the serum ox-LDL level was 52.860 mg/dL and the 25-(OH)-D concentration was 28.350 ng/L. In addition, there are certain differences in hematoma volume and fibrinogen among patients with different levels of ox-LDL and 25-(OH)-D. The inflammatory reaction triggered by the rise in ox-LDL and the fall in 25-(OH)-D levels may be linked to this, as it may lead to coagulation problems and hematoma growth. However, the specific mechanism still needs further analysis [27][28][29][30].

To clarify the relationship between serum ox-LDL levels and 25-(OH)-D and thereby analyse their interaction mechanism. Serum ox-LDL was negatively correlated with the level of 25-(OH)-D (P<0.05), indicating that there was also a certain relationship between serum ox-LDL and 25-(OH)-D. On the one hand, an elevated level of 25-(OH)-D can inhibit the release of proinflammatory factors, reduce the accumulation of low-density lipoprotein on the vascular wall, and thereby lower ox-LDL levels [31][32][33]. On the other hand, 25-(OH)-D can stimulate adiponectin release, which has anti-infection and lipid-regulating effects. It can promote fatty acid oxidation, thereby reducing lipid markers such as total cholesterol, triglycerides, and ox-LDL. Serum 25-(OH)-D levels and dyslipidemia in elderly patients with essential hypertension were examined in a prior study [34], which found that raising 25-(OH)-D levels could significantly improve several lipid markers. Another study [35] revealed that Triglycerides and serum vitamin D levels were inversely connected in individuals with type 2 diabetes (P<0.05).

## Conclusion

Before minimally invasive hematoma evacuation in patients with HICH complicated with cerebral oedema, the serum ox-LDL level was high, and the 25-(OH)-D level was low. Preoperative serum ox-LDL and 25-(OH)-D levels have high predictive value for cerebral oedema, and serum ox-LDL is closely related to 25-(OH)-D.

## Dodatak

### Funding

None.

### Ethical approval

This study was approved by the Medical Research Ethics Committee of our hospital (No. CAMS-CH-2022-022).

### Conflict of interest statement

All the authors declare that they have no conflict of interest in this work.
